# Improvement of Severe Volume Dependent Airway Closure With Tezepelumab: A Case Report

**DOI:** 10.1002/ccr3.73330

**Published:** 2026-08-12

**Authors:** Philipp Suter, Robert Greig, Rory Chan, Brian J. Lipworth

**Affiliations:** ^1^ Scottish Centre for Respiratory Research, School of Medicine University of Dundee Dundee UK

**Keywords:** asthma, spirometry, tezepelumab, type 2 inflammation

## Abstract

Severe asthma may be complicated by airway remodeling, manifesting as higher symptom burden, airflow limitation and reduced treatment response. The effect of biologics on lung function changes remains uncertain. We report a 31‐year‐old man with severe uncontrolled asthma and chronic rhinosinusitis with nasal polyposis, characterized by frequent exacerbations, marked type 2 inflammation, and pronounced volume‐dependent airway closure on spirometry with evidence of small airway dysfunction. Despite optimized high‐dose inhaled therapy, disease control remained poor. Treatment with tezepelumab was initiated following multidisciplinary assessment. After 12 months, the patient achieved normalization of symptoms, biomarkers, lung function, and flow–volume loop morphology without any further exacerbations. This case highlights a striking functional recovery associated with thymic stromal lymphopoietin blockade and suggests a potential role for tezepelumab in modifying early airway remodeling beyond symptom control.

## Key Clinical Message

Tezepelumab led to near normalization of lung function, flow–volume loop and symptom improvement with suppression of type 2 biomarkers. Furthermore, findings suggest TSLP inhibition might influence early airway remodeling.

## 1 Introduction

Asthma is a chronic inflammatory airway disease, marked by cough, dyspnoea, wheezing, and chest tightness. It is considered severe and uncontrolled when patients exhibit poor symptom control and experience recurrent exacerbations requiring oral corticosteroids despite optimized treatment and exclusion of alternative diagnosis [1].

However, the clinical expression of severe asthma on environmental insults including pathogens, allergens and pollutants is highly heterogeneous, as are its functional and radiological consequences. Some patients despite significant symptom burden, maintain near normal lung function and unremarkable imaging, whereas others may show pronounced airway remodeling on lung function testing [1, 2].

Airway remodeling refers to structural changes of both large and small airways resulting from unresolved persistent type 2 inflammation. However, direct mechanical stress present during bronchoconstriction through compression, shear stress or genetic and epigenetic alterations to epithelial components may initiate airway remodeling [2, 3, 4, 5, 6].

These alterations include epithelial cell dysfunction and apoptosis, goblet cell hyperplasia, airway smooth muscle (ASM) proliferation and fibroblast activation, concurrently leading to modifications in the cellular and extracellular matrix composition [3, 7]. The resultant changes within the bronchial walls and surrounding tissues including the vasculature ultimately narrow airway caliber and increase stiffness, manifesting as airflow limitation and worsening of respiratory symptoms. Airway remodeling has also been associated with consequent lack of treatment response [3]. On lung function testing airway remodeling is evident as reduced forced capacity along with marked volume dependent airway closure on the flow volume loop.

Biologic therapies modulating the underlying inflammatory process of the remodeling such as interleukin (IL) 4, IL‐5 and IL‐13 as well as the epithelial cell‐derived alarmins like thymic stromal lymphopoietin (TSLP) and IL‐33 may contribute to reversing airway remodeling [2, 3, 8].

## 2 Case History/Examination

A male in his 30s was referred in 2023 to the united airway clinic with a history of chronic rhinosinusitis with nasal polyposis (CRSwNP) since 2009 and severe uncontrolled asthma. He had never smoked tobacco or cannabis and there was no other notable medical history. His medication consisted of an as needed salbutamol inhaler and mometasone nasal spray. Although prescribed beclomethasone, he was not adhering to this. He reported several asthma exacerbations in the last year and persistent symptoms of chronic cough and dyspnoea.

## 3 Differential Diagnosis, Investigations and Treatment

**FIGURE 1:**
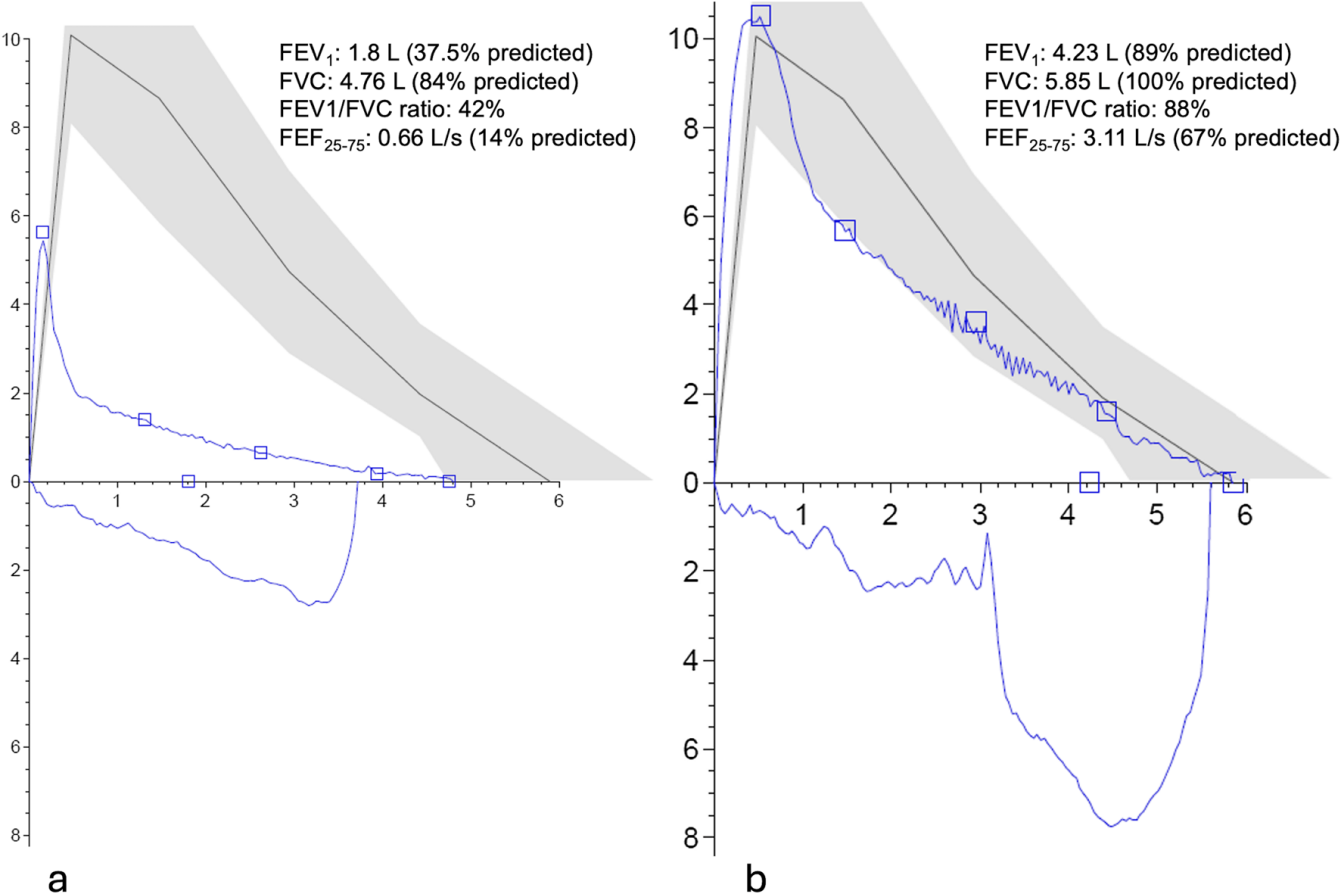
Flow‐volume loops (a) at baseline and (b) after 1 year of tezepelumab treatment FEV_1_, forced expiratory volume in 1 , FVC, forced vital, FEF_25‐75_, forced mid‐expiratory flow.

Serum biomarkers demonstrated marked type 2 high inflammation, with a blood eosinophil count (BEC) of 580 cells/μL, total IgE level of 669 kU/L with raised specific IgE levels to grass and birch pollen as well as to cat and dogs. Fractional exhaled nitric oxide (FeNO) value was 84 ppb. Spirometry revealed an obstructive defect with a severely scalloped flow–volume loop; no bronchodilator reversibility was performed (Figure 1a). The forced expiratory volume in 1 s (FEV_1_) was 1.8 L (% predicted 37.5%), the forced vital capacity (FVC) 4.76 L (% predicted 81%), FEV_1_/FVC ratio 42% and forced mid‐expiratory flow (FEF_25‐75%_) 0.66 L/s (14% predicted). In addition, oscillometry indicated small airway dysfunction, with abnormal values for peripheral resistance (R5–R20) of 0.13 kPa/L/s and peripheral compliance (AX) of 1.26 kPa/L/s.

He also reported anosmia and obstructed nasal breathing, albeit a low Sino‐Nasal Outcome Test (SNOT22) score of 18/110. A diagnosis of chronic rhinosinusitis with nasal polyposis was confirmed with rigid nasal endoscopy, which revealed mucosal swelling and a nasal polyp score of 2 out of 8. CT showed mucosal thickening in bilateral maxillary and right frontal sinuses, while HRCT showed no wall thickening or mucus plugging. Initial treatment was with budesonide/formoterol, which was escalated to high‐dose beclomethasone/formoterol/tiotropium, as well as montelukast and cetirizine.

Despite 4 months of optimal treatment, the patient continued to have poor symptom control, reflected by an asthma control questionnaire (ACQ) score of 1.8 with three further severe exacerbations requiring oral corticosteroids. He was then referred for multidisciplinary team discussion where treatment with tezepelumab was instigated, which was subsequently well tolerated.

## 4 Conclusion and Results (Outcome and Follow‐Up)

During follow up, the patient experienced substantial clinical improvements without any further exacerbations. After 1 year of tezepelumab, the ACQ score decreased to 0 and a SNOT22 score to 2 (normal < 10). Biomarker levels (BEC 80 cells/μL, total IgE 586 kU/L and FeNO 16 ppb), and lung function including flow‐volume loop showed near normalization (Figure 1b), including dynamic lung volumes (FEV_1_ 4.23 L (89%), FVC 5.85 L (100%), FEV_1_/FVC 88%, FEF_25‐75_ 3.11 L/s (67%)). Additionally, repeat nasal endoscopy revealed no nasal polyps. Because of this substantial improvement, the patient was stepped down to beclomethasone/formoterol as a maintenance and reliever therapy while stopping tiotropium.

## 5 Discussion

This case highlights dramatic physiological improvements in a patient with severe uncontrolled asthma following treatment with tezepelumab. The degree of airflow limitation was pronounced, including substantial small airway disease, as reflected by the markedly reduced FEF_25‐75%_ and impaired peripheral resistance and compliance, despite an absence of wall thickening or mucus plugging on imaging. When first seen in clinic the impression was of typical changes of airway remodeling on lung physiology testing and the patient was accordingly counseled regarding the possibility of limited improvement in response to tezepelumab. To our knowledge, no similar cases have been reported in the literature.

The marked improvement in lung physiology in our case suggests that upstream biologic therapies such as tezepelumab may potentially influence early airway remodeling, which was previously considered to be less modifiable [3]. By targeting epithelial alarmin pathways influencing downstream type 2 cytokines, such therapies may influence key structural and inflammatory interactions, including interactions between epithelial cells, immune cells such as macrophages and fibroblasts, as well as signaling between mast cells and ASM cells [9]. This wide action is especially important as endotypes and phenotypes may overlap during a patient's lifetime [3]. TSLP induces the release of angiogenic and lymphangiogenic factors in macrophages and promotes the activation of fibroblasts which could be decreased by tezepelumab [2, 10, 11].

However, in the CASCADE study, no significant differences at bronchial biopsy were observed in airway submucosal cells other than eosinophils. Furthermore, histopathological assessment revealed no changes in reticular basement membrane thickness and epithelial integrity between the tezepelumab and placebo groups. Although CT imaging demonstrated an increased lumen area across airway generations in patients receiving tezepelumab, this finding might reflect reduced mucus plugging rather than a true attenuation of remodeling. It is important to note that this study enrolled patients with moderate to severe asthma, whose baseline histopathological and radiological characteristics were closer to those of healthy controls than to those typically observed in severe asthma [12]. This is in contrast to studies using murine asthma models where TSLP antibodies achieved inhibition in airway remodeling [2]. Pointedly, in CASCADE no improvements were seen in either FEF_25‐75_, peripheral resistance or compliance [12].

There are several limitations in this case report. First, bronchodilator reversibility testing was not performed at baseline, which may have helped to identify reversible airflow obstruction as a potential treatable trait. Second, body plethysmography was not available, precluding assessment of static lung volumes and potential changes in air trapping and hyperinflation, which may have been relevant in this patient with severe asthma.

Although true reversal of structural airway changes cannot be confirmed without histopathological evidence, the substantial improvements in lung function, symptoms, and reduced treatment use support the hypothesis that TSLP inhibition may favorably impact established airway remodeling. These findings suggest a role for TSLP blockage in modifying disease trajectory beyond symptom control in patients with airway remodeling.

## Author Contributions

**Philipp Suter:** writing – original draft, methodology, visualization, data curation. **Robert Greig:** writing – review and editing. **Rory Chan:** writing – review and editing. **Brian J. Lipworth:** writing – review and editing, methodology, validation, supervision.

## Funding

The authors have nothing to report.

## Ethics Statement

The authors have nothing to report.

## Consent

The authors declare that written informed consent was obtained for the publication of this manuscript and accompanying images using the consent form provided by the Journal.

## Conflicts of Interest

Philipp Suter reports a relationship with AstraZeneca UK Limited that includes: speaking and lecture fees. Philipp Suter reports a relationship with GSK that includes: speaking and lecture fees. Philipp Suter reports a relationship with Lung League Fribourg (Switzerland) that includes: funding grants without influence on work reported in this paper. Philipp Suter reports a relationship with Swiss Lung Foundation (Switzerland) that includes: funding grants without influence on work reported in this paper. Robert Greig reports a relationship with AstraZeneca UK Limited that includes: speaking and lecture fees and support attending BTS. Rory Chan reports a relationship with AstraZeneca UK Limited that includes: speaking and lecture fees and travel reimbursement. Rory Chan reports institutional grants awarded from Asthma+Lung UK, Chiesi, AstraZeneca and GSK; serving on advisory boards for AstraZeneca and Vitalograph; personal fees (talks and/or drafting educational material) from AstraZeneca, Chiesi, Thorasys and Vitalograph; and support attending meetings from AstraZeneca, Chiesi, NIOX, Sanofi‐Regeneron and Vitalograph. Brian J. Lipworth reports a relationship with AstraZeneca UK Limited that includes: consulting or advisory, funding grants, speaking and lecture fees, and travel reimbursement. Brian J. Lipworth reports a relationship with GSK that includes: non‐financial support. Brian J. Lipworth reports a relationship with Sanofi that includes: speaking and lecture fees. Brian J. Lipworth reports a relationship with Circassia Pharmaceuticals Plc that includes: consulting or advisory and speaking and lecture fees. Brian J. Lipworth reports a relationship with Teva UK Ltd. that includes: consulting or advisory, funding grants, speaking and lecture fees, and travel reimbursement. Brian J. Lipworth reports a relationship with Chiesi Ltd. that includes: consulting or advisory, funding grants, speaking and lecture fees, and travel reimbursement. Brian J. Lipworth reports a relationship with Lupin Healthcare UK Ltd. that includes: consulting or advisory. Brian J. Lipworth reports a relationship with Glenmark Pharmaceuticals Limited that includes: consulting or advisory. Brian J. Lipworth reports a relationship with Dr. Reddy's Laboratories Ltd. that includes: consulting or advisory. Brian J. Lipworth reports a relationship with Sandoz UK Ltd. that includes: consulting or advisory. Brian J. Lipworth reports a relationship with Boehringer Ingelheim Ltd. that includes: consulting or advisory, speaking and lecture fees, and travel reimbursement. Brian J. Lipworth reports a relationship with Mylan Pharmaceuticals Inc. that includes: consulting or advisory and speaking and lecture fees. The son of Dr. Brian Lipworth is presently an employee of AstraZeneca.

## Data Availability

The data that support the findings of this study are available on request from the corresponding author.
